# Off-the-shelf multi-branch endograft for the treatment of chronic type B aortic dissection with prior thoracic endovascular aortic repair

**DOI:** 10.1016/j.jvscit.2025.101881

**Published:** 2025-06-17

**Authors:** Dai Yamanouchi, Courtney Morgan, Elise DeRoo, Elizabeth Yates

**Affiliations:** a University of Wisconsin School of Medicine and Public Health, Madison, WI; b Department of Vascular Surgery, Fujita Health University, Toyoake, Japan

**Keywords:** Type B aortic dissection, Thoracoabdominal aortic aneurysm, Branched endovascular aortic repair, Off-the-shelf multi-branched stent grafts, The PETTICOAT stent

## Abstract

We report a case of a 51-year-old male with a history of chronic type B aortic dissection status post thoracic endovascular aortic repair who presented with a progressive thoracoabdominal aortic aneurysm. The patient underwent a successful off-the-shelf multibranch stent graft procedure for complete sealing of the false lumen, with extensions into visceral and renal arteries. This case highlights the advantages of this technique, offering comprehensive sealing of entry tears and enhanced false lumen thrombosis. Preoperative computed tomography imaging, intraoperative angiographic images, and postoperative computed tomography findings are presented to illustrate the procedure’s effectiveness and clinical outcomes.

Chronic type B aortic dissection (cTBAD) with associated thoracoabdominal aortic aneurysm (TAAA) presents a complex therapeutic challenge.^1^, ^2^, ^3^, ^4^, ^5^, ^6^ Various endovascular techniques have been developed, including false lumen embolization, candy plug, Provisional Extension To Induce Complete Attachment (PETTICOAT), and Knickerbocker procedures.^7^, ^8^, ^9^, ^10^, ^11^ These approaches aim to stabilize the aorta by inducing thrombosis of the false lumen while maintaining perfusion of branch vessels. However, persistent flow in the false lumen often necessitates reintervention. The off-the-shelf multibranch endograft, Gore Excluder thoracoabdominal branch endoprosthesis (TAMBE), offers a novel solution by providing complete sealing of the false lumen while preserving visceral and renal perfusion.^12^ Here, we present a case demonstrating the efficacy of TAMBE in managing cTBAD with prior thoracic endovascular aortic repair (TEVAR).

## Case presentation

A 51-year-old male with a history of motor vehicle accident, aortic dissection, and stroke was referred for evaluation of a progressive TAAA. The patient had undergone TEVAR at an outside hospital 4 years prior with placement of a Cook dissection stent (30 × 30 × 142 mm) and a bare metal dissection stent (36 × 180 mm) and carotid-subclavian bypass with left subclavian plug.

**Fig 1:**
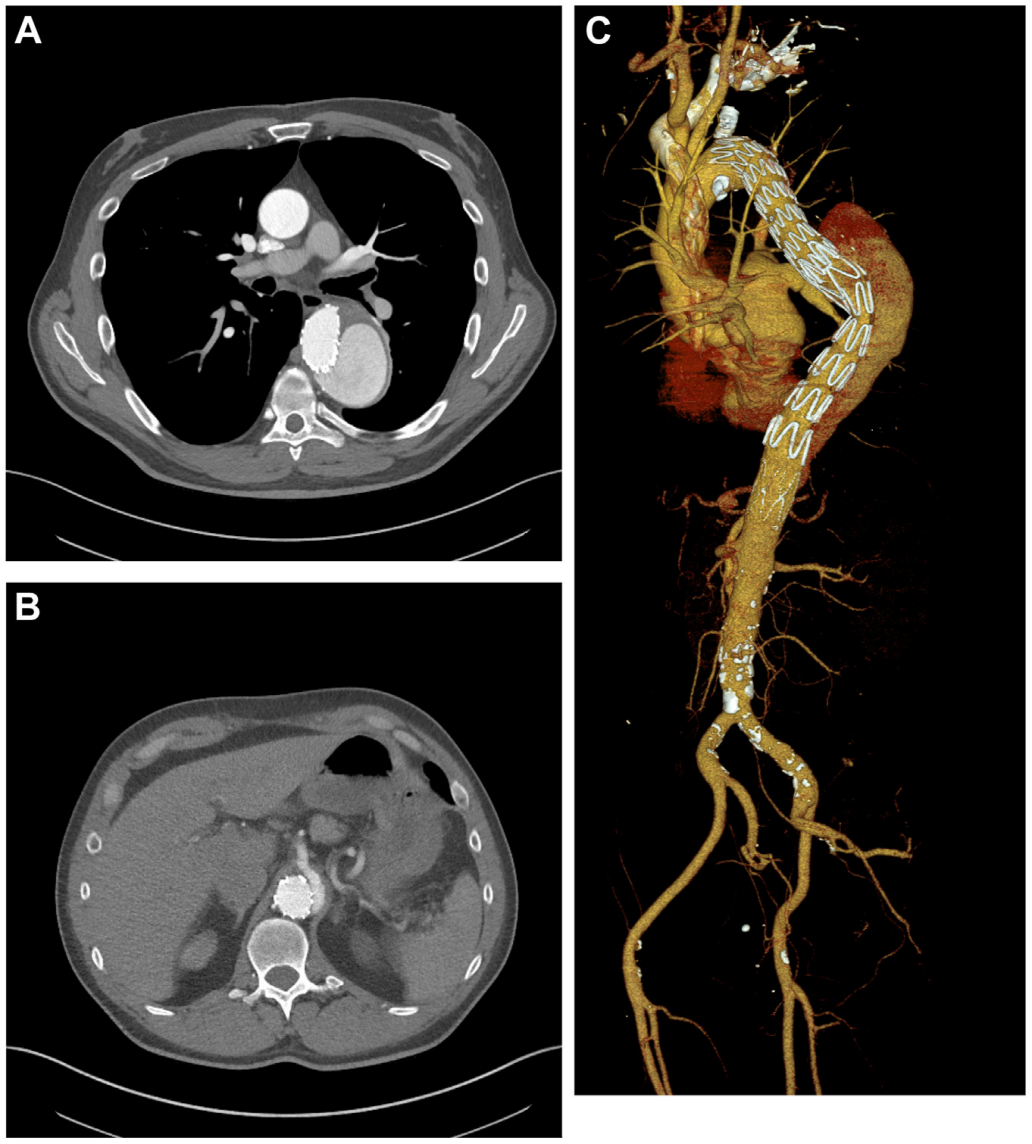
Preoperative images. **(A)** Axial image of aneurysm at the descending aorta with prior thoracic endovascular aortic repair (TEVAR) graft. **(B)** Bare-metal stent at the level of the celiac artery with chronic dissection. **(C)** Three-dimensional reconstructed image of the computed tomography angiogram (CTA).

Computed tomography angiogram (CTA) revealed a chronic type B_3,6_ aortic dissection (cTBAD) and type I TAAA measuring 6.3 cm (Supplementary Fig 1, online only). False lumen perfusion with multiple entry tears and flap extension into the celiac was noted. Branch vessels, including the superior mesenteric artery (SMA) and renal arteries, were patent (Fig 1). The celiac artery was fed by the false lumen, whereas the SMA and renal arteries were fed by the true lumen, with the dissection flap terminating in the visceral segment (Supplementary Fig 2, online only).

Given the patient’s cTBAD, aneurysmal degeneration, and risk of rupture, we discussed treatment options including open thoracoabdominal repair, candy plug, false lumen embolization, and multibranch stent graft placement. The patient opted for a multibranch stent graft procedure because of its minimally invasive nature and ability to achieve complete false lumen exclusion.

**Fig 2:**
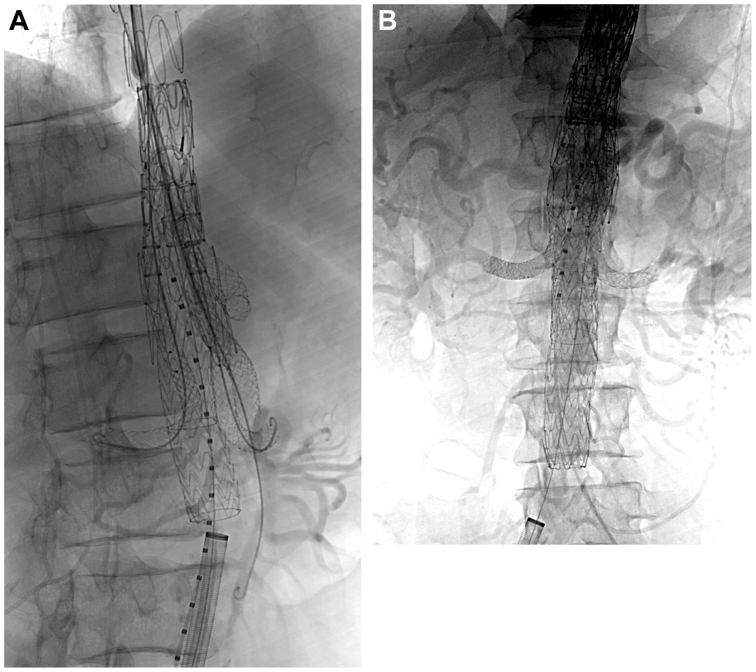
Intraoperative images. **(A)** Placement of multi side branch endograft and branch stent graft with in the previously placed bare-metal stent. **(B)** Completion angiogram.

The TAMBE procedure was performed under general anesthesia with spinal cord protection. Following spinal drain placement and sterile preparation, access was achieved via surgical cutdown of the right axillary artery and percutaneous punctures of the femoral arteries. A 37-mm TAMBE stent graft was deployed from the descending thoracic aorta to the diaphragmatic hiatus. Intentional catheter selection allowed cannulation of the dissected celiac, which required traversing the previously placed bare-metal stent from the true lumen into the celiac origin off the false lumen. Each portal was bridged with VBX stent grafts (celiac, 9 × 59 mm; SMA, 9 × 79 mm; left renal, 7 × 79 and 7 × 59 mm; right renal, 7 × 59 mm), with size determined by a diameter 1- to 2-mm greater than the target vessel and the length chosen to include at least a 20-mm landing zone in the target vessel. Extensions were added proximally with a 37 mm × 20 cm GORE TAG conformable thoracic stent graft and distally with a 26 mm × 10 cm conformable thoracic stent graft to exclude the aneurysm and achieve a distal seal within nonaneurysmal aorta. Molding balloons were used at all junctions. Completion angiography confirmed branch patency and no evidence of type I or III endoleak, although a small type II endoleak was noted at T9 (Fig 2). The patient tolerated the procedure well with no complications.

Per our institutional protocol, the patient was admitted to the intensive care unit. He was passively rewarmed and extubated on the evening of surgery. On the first postoperative day, the patient had no evidence of paralysis, and the spinal drain was removed. Over the subsequent 2 days, the patient tolerated a diet and ambulated easily, only complaining of an intermittent, nonpositional headache. He was discharged home off his baseline Xarelto on the fourth postoperative day with steady improvement in his headache. He resumed his Xarelto on postoperative day 9 after near resolution of his headaches.

**Fig 3:**
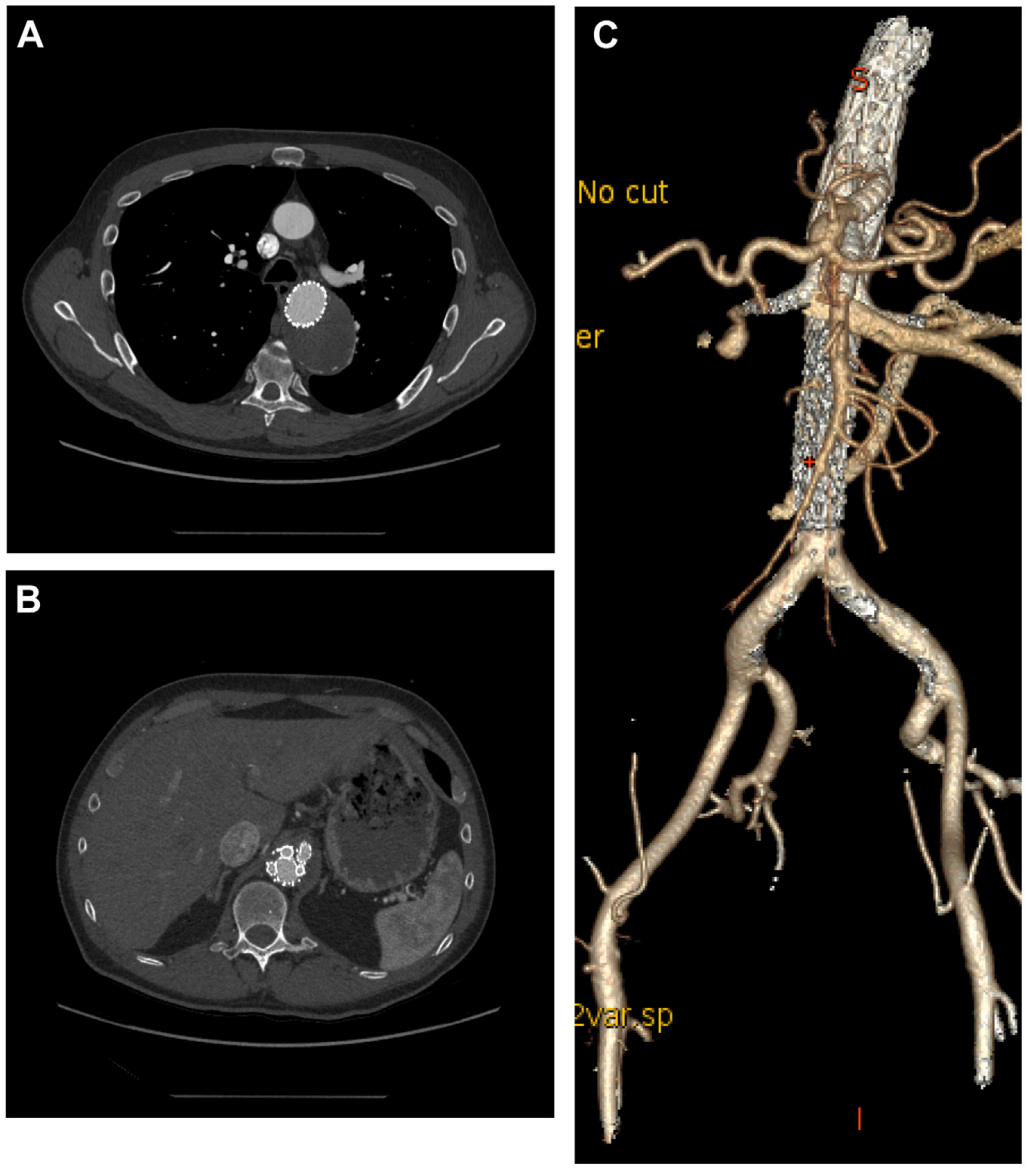
Postoperative images. **(A)** Complete thrombosis of the aneurysm at the proximal descending aorta. **(B)** Multi-branch stent graft at the level of the celiac artery. **(C)** Three-dimensional (3D) reconstructed image of the computed tomography (CT) angiogram.

One-month postoperative CTA demonstrated patent stent grafts with exclusion of the false lumen (Fig 3). The aneurysm sac remained stable in size, and there was no evidence of type I or III endoleak. A persistent small type II endoleak was noted, likely arising from a lumbar artery. He was scheduled for follow-up CTA in 1 year.

Written informed consent was obtained from the patient for the publication of this report, including all relevant clinical details and accompanying images.

## Discussion

This case highlights the effectiveness of off the shelf multibranch endograft placement for cTBAD. Given that the dissection extended into the thoracoabdominal region, involving critical re-entry tears around the celiac artery, and with the PETTICOAT stent extending to the SMA, TAMBE was selected for its ability to provide definitive sealing of the entry tear. Compared with alternatives such as candy plug, PETTICOAT, or Knickerbocker techniques, TAMBE achieves definitive exclusion of entry tears while minimizing procedural complexity, and stabilizes the aorta more effectively than techniques relying solely on proximal sealing or adjunct embolization.^2^^,^^7^, ^8^, ^9^^,^^12^ The effectiveness of TAMBE is particularly evident in cases with complex anatomy or extensive dissection flaps involving visceral and renal arteries.^12^, ^13^, ^14^ Traditional techniques like the candy plug and PETTICOAT may leave residual perfusion in the false lumen, increasing the risk of continued aortic expansion.^7^^,^^8^ Similarly, although Knickerbocker and false lumen embolization techniques have shown success in select patients, they require meticulous planning and may not provide the same comprehensive exclusion of the false lumen as TAMBE.^8^ Importantly, TAMBE can be easily extended proximally or distally to treat different extent TAAAs or TBADs. In this case, we extended proximally and distally with TEVARs to cover the uncovered dissection stent graft and land in the healthy aorta. With nonaneurysmal iliac arteries bilaterally, there was no need to add complexity and time to the case by deploying stent grafts that sealed in the iliac arteries.

The off-the-shelf nature of TAMBE is also an advantage. The graft is readily available without the need for creation of fenestrations, in contrast to physician-modified endografts (PMEGs).^15^ The use of an off-the-shelf device facilitates timely intervention in urgent or elective settings, eliminating the delay associated with custom-made grafts. Despite their time disadvantages in urgent settings, PMEGs have been utilized to treat TAAAs and specifically TAAAs associated with cTBAD.^16^, ^17^, ^18^ In the future, studies comparing TAMBE and PMEGs for the treatment of cTBAD-associated TAAAs should be performed to assess the efficacy of both approaches. Beyond endovascular options, open repair should be considered for patients who could tolerate the physiologic stress of an open TAAA repair, but shared decision-making that takes into account patient preferences should be performed.

We acknowledge that TAMBE has been described to treat chronic post dissection aneurysms.^18^ However, this utilization is off the instructions for use in a device that was only recently approved for clinical use. Moreover, it has not yet been described in the literature navigating around a dissection stent to deploy visceral branches. This case demonstrates that the dissection stent is not a barrier to TAMBE and that the portal branches can be delivered and deployed without kinking, crushing, or adverse early outcomes.

Despite its benefits, TAMBE is not without limitations. Postoperative minor endoleaks may necessitate more frequent surveillance. Moreover, although our 30-day data is encouraging, in the future, data about the long-term durability of TAMBE in dissection should be collected across multiple patients.

## Conclusions

The off-the-shelf TAMBE stent graft is an effective option for managing TBAD with aneurysmal degeneration. Its ability to achieve complete false lumen exclusion while maintaining visceral artery patency demonstrates its potential as a preferred strategy in complex aortic pathologies.

## Funding

None.

## Disclosures

D.Y. reports an educational grant to the University of Wisconsin from W. L. Gore & Associates and a research grant to Fujita Health University from W. L. Gore & Associates.
