# Supplementary figures and images for: Off-the-shelf multi-branch endograft for the treatment of chronic type B aortic dissection with prior thoracic endovascular aortic repair
Source: J Vasc Surg Cases Innov Tech. 2025 Jun 17;11(5):101881. doi: 10.1016/j.jvscit.2025.101881 (PMC12275863; doi:10.1016/j.jvscit.2025.101881)

## Slide 1
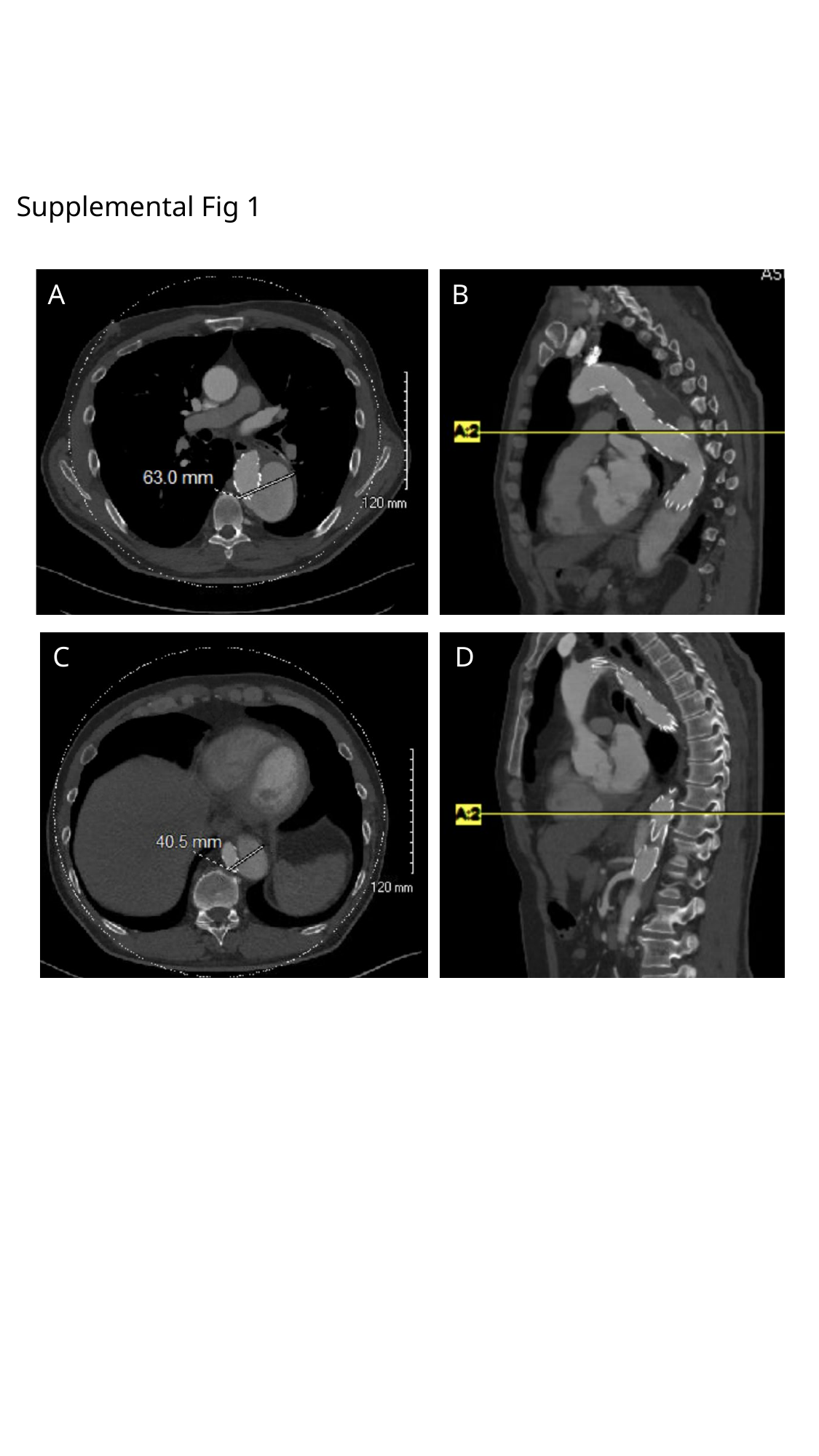

Supplemental Fig 1
A
B
C
D

## Slide 2
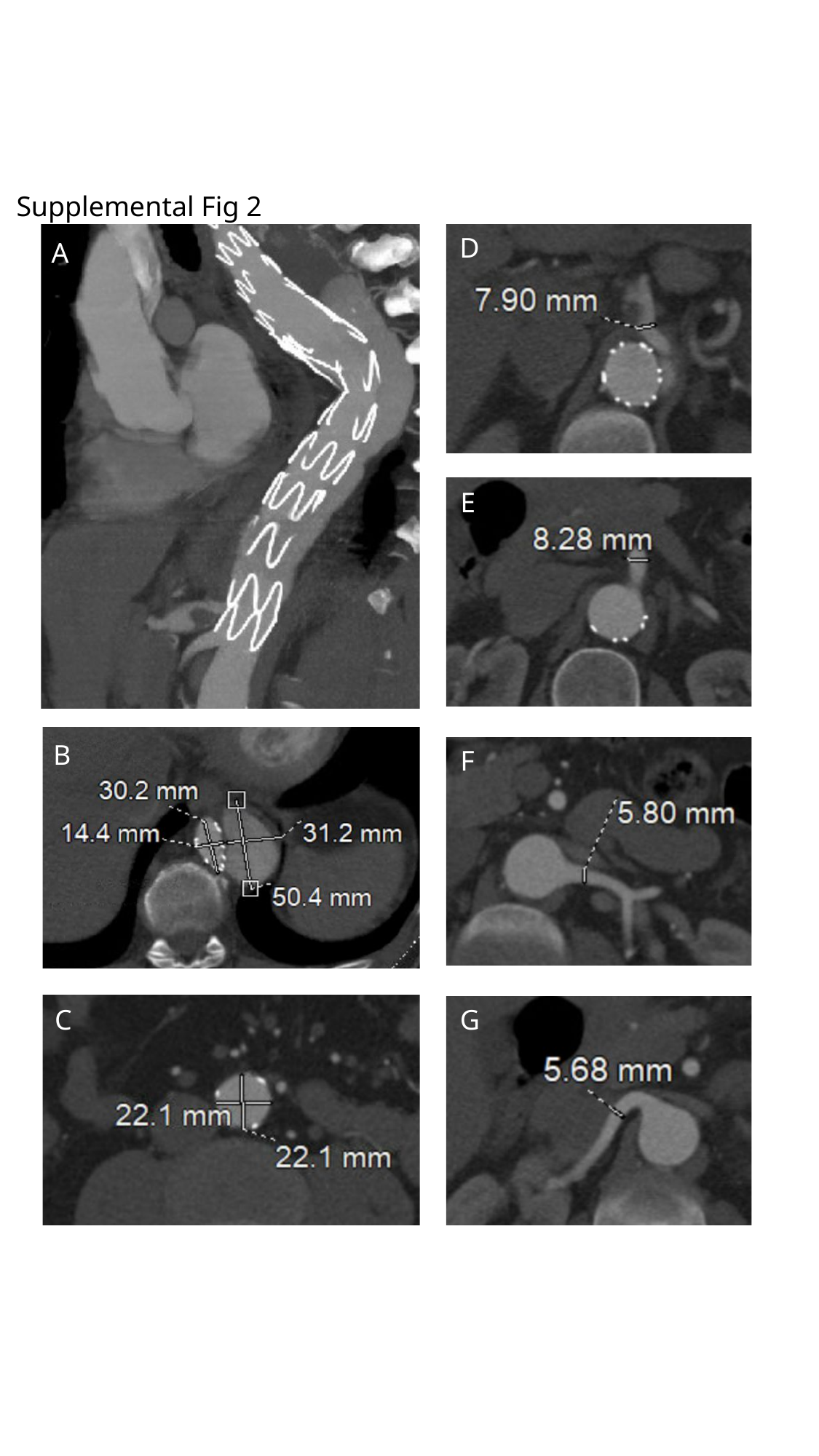

Supplemental Fig 2
D
A
E
B
F
G
C

Supplement: Supplementary Material [file mmc1.pptx]
